# Low-replication influenza virus mediates high pathogenicity through an inflammation-driven lung–heart–brain axis in mice

**DOI:** 10.1080/22221751.2025.2608406

**Published:** 2026-01-07

**Authors:** Wenfei Zhu, Zhuoya Xu, Xinglian Wang, Xiyan Li, Zi Li, Guolin Dong, Lei Yang, Ye Zhang, Ruina You, Yousong Peng, Dayan Wang

**Affiliations:** a NHC Key Laboratory of Medical Virology and Viral Diseases, National Institute for Viral Disease Control and Prevention, Chinese Center for Disease Control and Prevention (CDC), National Key Laboratory of Intelligent Tracking and Forecasting for Infectious Disease, Beijing, People’s Republic of China; b Bioinformatics Center, College of Biology, Hunan Provincial Key Laboratory of Medical Virology, Hunan Research Center of the Basic Discipline for Cell Signaling, Hunan University, Changsha, People’s Republic of China

## Abstract

The outcomes of viral infections typically correlate with viral load in host tissues. In this study, we identified a H3N2 strain A/Environment/Guangxi/44461/2019 (GX19) that induced rapid mortality in mice by 4 days post-infection despite exhibiting low pulmonary replication capacity. Pathological analysis revealed that GX19 at 10^6^ TCID_50_ (GX19-6) caused more severe lung damage than GX19 at 10^5^ TCID_50_ (GX19-5), while inducing pulmonary pathology comparable to a H3N8 virus A/Changsha/1000/2022 at 10^6^ TCID_50_ (CS-6). Both GX19-6 and CS-6 triggered greater cardiac damage than GX19-5. Notably, GX19-6 displayed unique neurovirulence, eliciting significantly more severe brain damage than GX19-5 and CS-6, accompanied by evident cerebral haemorrhage. Gene Set Variation Analysis (GSVA) revealed distinct cardiac gene expression profiles among viral infections. Specifically, GX19-5 up-regulated gene sets associated with arrhythmia, whereas GX19-6 triggered pathways involved in cardiac arrest. Neither of these effects was present in CS-6 infection. In the brain, GX19-6 specifically induced stronger upregulation of cerebral venous thrombosis and acute ischaemic stroke gene sets compared to other groups, consistent with its pronounced neuropathology. Transcriptomic profiling demonstrated significant alterations across all three organs in GX19-6-infected mice, showing suppression of T-cell immunity in the lungs and brain alongside elevated systemic inflammation. In the heart, increased inflammation and apoptosis were accompanied by impaired energy metabolism and reduced cardiac function, potentially contributing to the observed hypoxic responses in the heart, lungs, and brain. Collectively, these findings reveal an inflammation-driven lung-heart–brain axis in influenza virus pathogenicity.

## Introduction

The influenza virus is a major infectious agent posing significant threats to global public health. Historically, influenza pandemics have caused catastrophic mortality, as exemplified by the 1918 Spanish flu pandemic, which claimed at least 50 million lives worldwide [1,2]. Seasonal influenza viruses infect approximately a billion people annually, including 3–5 million cases of severe illness [3]. The virus primarily invades the host through the respiratory tract, with clinical outcomes ranging from asymptomatic infection or mild respiratory illness to severe systemic disease and fatal outcomes [4,5]. This broad spectrum of disease severity stems from complex virus-host interactions: viral pathogenicity is determined by immune evasion mechanisms (e.g. NS1 protein-mediated suppression of host antiviral responses) and replication efficiency, while host susceptibility depends on the robustness of innate and adaptive immune defences [6-8].

Numerous studies have shown that influenza virus infections extend beyond pulmonary involvement, triggering systemic effects that can induce neuroinflammation and cardiac injury[9,10]. The severity of neurological complications caused by influenza infection varies. Mild cases present with confusion and severe headache, while severe cases may develop into conditions such as encephalitis and Guillain-Barré syndrome[11,12]. Experimental studies in murine models have demonstrated that even in the absence of detectable live virus in brain tissue, influenza virus infections can activate microglia and astrocytes and lead to elevated levels of pro-inflammatory cytokines[13,14]. Epidemiological studies have established a clear association between influenza virus infection and an increased risk of acute myocardial infarction, myocarditis, and heart failure[9,15-17]. Consistent with this, clinical observations frequently report elevated cardiac biomarkers (e.g. cardiac troponin) and electrocardiographic abnormalities during influenza virus infections[18,19]. Further studies using mouse models have shown that the myocardial inflammation and functional abnormalities caused by viral infection are mainly a type of immune pathological damage mediated by an excessive immune response[20].

Typically, higher viral replication correlates with increased tissue damage and worse clinical outcomes [21,22]. Intriguingly, we identified a distinct viral strain with low replicative potential but pronounced pathogenicity during studies of the biological features of H3 subtype influenza viruses in a mouse model. Subsequent typing confirmed this strain as H3N2, which was isolated from live poultry-related environments. In an effort to uncover the molecular mechanisms underlying its unique “low replication but high pathogenicity” phenotype, we conducted a series of experiments, encompassing assessments of tissue tropism, replication ability, histopathological alterations, as well as transcriptome sequencing. The result revealed that this strain may drive lethal outcomes through a previously unrecognized mechanism – a pathogenic lung-heart–brain axis that precipitates fatal disease independent of high viral titres.

## Results

### GX19 virus causes rapid death of mice with limited replication both *in vivo* and *ex vivo*

**Figure 1.**
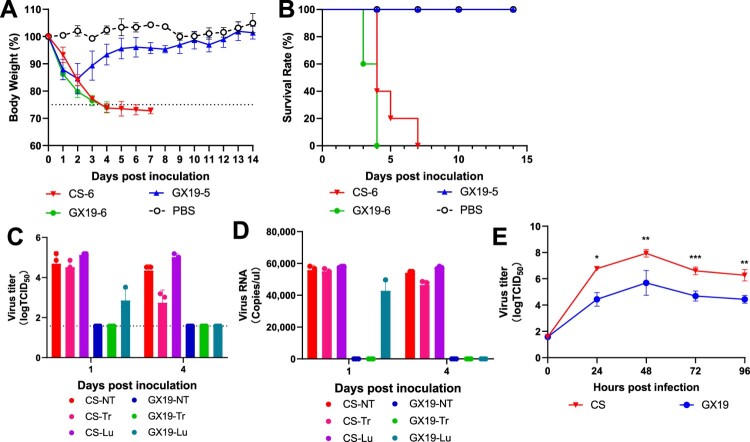
Morbidity, mortality, and replication of GX19 viruses. (A) Five 8-week-old C57BL/6 mice per group received intranasal inoculation of either 10^5^ or 10^6^ TCID_50_ of the indicated viruses. Daily body weight checks were conducted over a 14 dpi. Euthanasia was performed humanely on mice that experienced a ≥ 25% loss of their original body weight. (B) Depicts the survival rate percentages of C57BL/6 mice after exposure to the indicated viruses. (C and D) Six C57BL/6 mice were assigned to groups and given 10^6^ TCID_50_ of the designated viruses via intranasal administration. At 1 and 4 dpi, three mice from each group were euthanized. Respiratory tract tissues such as the nasal turbinate (NT), trachea (Tr), and lungs (Lu) were harvested individually for TCID_50_ testing (C) or RT-qPCR (D), respectively. (E) The growth characteristics of the GX19 and CS were compared in MDCK cells maintained at 37°C. The supernatants were collected, and the virus titres were determined using TCID_50_ in MDCK cells at the indicated time points. Results are expressed as the mean value plus or minus standard deviation (SD), with a sample size of 3 (n = 3). GX19, A/Environment/Guangxi/44461/2019 (H3N2). GX19-6 and GX19-5 represent 10^6^ and 10^5^ TCID_50_ of GX19 virus. CS, A/Changsha/1000/2022 (H3N8). CS-6 represents 10^6^ TCID_50_ of the CS virus. *, *p*-value <0.05; **, *p*-value <0.01; ***, *p*-value <0.001.

As shown in Figure 1A, when inoculated with viral strains A/Environment/Guangxi/44461/2019 (H3N2) or A/Changsha/1000/2022 (H3N8) at 10^6^ TCID_50_ (denoted as GX19-6 and CS-6, respectively), all mice died during the observation period. However, GX19-6 inoculated mice exhibited more body weight loss than CS-6 inoculated mice at 1 d post infection (dpi) and 2 dpi, with *p*-value <0.05 and *p*-value <0.01, respectively, and thus all 5 mice died 3 days earlier than those of CS-6. By comparison, body weight loss occurred at the first two days post inoculation but was continuously increasing at the following observation period for the mice inoculated with A/Environment/Guangxi/44461/2019 at 10^5^ TCID_50_ (denoted as GX19-5). The maximum body weight loss was 17%, and all mice survived. In accordance with the body weight change, 5/5, 5/5, and 0/5 mice were dead when inoculated with GX19-6, CS-6, and GX19-5, respectively (Figure 1B). Tissue tropism of both A/Environment/Guangxi/44461/2019 (GX19) and A/Changsha/1000/2022 (CS) was investigated by both TCID_50_ and RT-qPCR assays. Viruses can only be detected in respiratory tissues. Other tissues, including brains, heart, spleen, liver, eyes, kidneys, and intestinal tissues, were negative for influenza A viruses. Replication ability was also investigated both *in vivo* and *ex vivo* for both viral strains (Figure 1C-E). As expected, the CS exhibited robust replication both in respiratory tissues and the MDCK cell line. However, the GX19 virus can only be detected in lung tissue, but not in the nasal turbinate or trachea (Figure 1C&D). In addition, the viral load in lung tissue or MDCK cells for GX19 virus was significantly lower than that for CS viruses (*p*-value < 0.05 in the t-test). This result indicated that the GX19 virus causes rapid death of mice but with limited replication both *in vivo* and *ex vivo*.

### Pathological changes in the mouse lungs, hearts, and brains after viral infections

**Figure 2.**
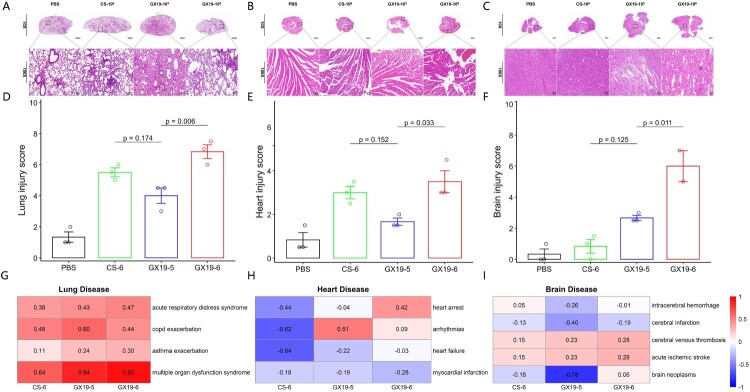
Pathological changes in the lung, heart, and brain of mice after viral infections. (A)-(C) refer to the images of mice's lungs, hearts, and brains, respectively, by H&E staining (10×, Scale bar = 1 mm for low magnification; 100×, Scale bar = 100 μm for high magnification). (D)–(F) refer to quantitative measures of injuries in mice's lungs, hearts, and brains, respectively (see Materials and Methods). *P*-values were shown for comparison between CS-6, GX19-5, and GX19-6 using the t-test. n = 3 mice per group except for the brain of the GX19-6 group, where n = 2 due to severe degradation of a mouse brain sample in the H&E experiment. (G)-(I) refer to GSVA results of acute diseases in the lungs, hearts, and brains, respectively.

Subsequent pathological examination of three major organs (lungs, heart, and brain) in GX19-6 infected mice revealed distinct tissue-specific damage patterns associated with the rapid mortality phenotype. Pulmonary histopathology (Figure 2A) demonstrated characteristic influenza-induced damage, with both GX19-6 and CS-6 infections causing severe oedema and inflammatory infiltration at comparable levels, significantly exceeding the moderate pathology observed in GX19-5 infected mice (Figure 2D & Table S1). Cardiac sections exhibited dose-dependent injury (Figure 2B&E and Table S2), where GX19-5 induced only focal inflammation and minor structural disruption, while both GX19-6 and CS-6 infections provoked extensive myocardial damage with dense inflammatory infiltrates. Most strikingly, neuropathological analysis (Figure 2C) uncovered GX19-specific virulence, with both doses inducing cerebral haemorrhage and oedema – effects absent in CS-infected mice. Quantitative assessment (Figure 2F & Table S3) confirmed this neurotropism, showing GX19-6-induced brain injury scores (only two brain samples for GX19-6 due to severe degradation of a mouse brain sample in the H&E experiment) nearly double those of GX19-5 (*p*-value <0.01), while CS-6 caused minimal neurological damage.

Transcriptomic profiling via RNA-Seq was conducted on lung, cardiac, and brain tissues to systematically characterize host responses to viral infection. Gene Set Variation Analysis (GSVA) of acute disease-associated pathways revealed organ-specific dysregulation patterns. Pulmonary transcriptomes showed significant upregulation of gene sets related to acute respiratory distress syndrome (ARDS), COPD exacerbation, asthma exacerbation, and multiple organ dysfunction syndrome across all three viral infections (GX19-5, GX19-6, and CS-6), with no virus-specific differences in magnitude. Cardiac analysis demonstrated distinct viral strain-dependent effects: while CS-6 infection down-regulated gene sets associated with cardiac arrest, arrhythmias, heart failure, and myocardial infarction, GX19-6 uniquely activated gene sets associated with heart arrest, and GX19-5 specifically induced upregulation of arrhythmia-related gene sets. Cerebral transcriptional changes were particularly striking, with evaluation of five neurological disease pathways (intracerebral haemorrhage, cerebral infarction, cerebral venous thrombosis, acute ischaemic stroke, and brain neoplasms) revealing that GX19-6 infection induced the most pronounced upregulation of cerebral venous thrombosis and acute ischaemic stroke gene sets – a finding consistent with the severe haemorrhagic pathology observed in GX19-6 infected brains. These results demonstrate that while all three virus infections induce acute pulmonary injury pathways, GX19-6 drives unique transcriptional programmes in both cardiac and neural tissues that correlate with its distinct pathological manifestations.

### Transcription analysis of GX19-6 infection

**Figure 3.**
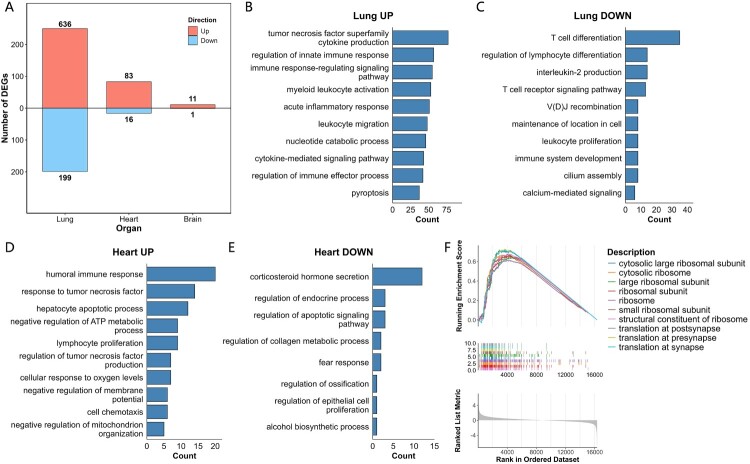
Transcription analysis of GX19 infection. (A) Number of DEGs identified in the lung, heart, and brain. (B)∼(E) refer to the enriched biological processes of up - and down-regulated DEGs in the lung and heart after infection with GX19-6. The GO terms were clustered, and each subpanel showed the number of GO terms in each cluster. (F) Top 10 enriched KEGG pathways in the mice brain by GSEA analysis.

To elucidate the molecular mechanisms of GX19 pathogenicity in mice, we performed a comprehensive transcriptomic analysis. Lung tissue profiling revealed 835 differentially expressed genes (DEGs) post GX19-6 infection when compared to the PBS group (636 up-regulated and 199 down-regulated; Figure 3A). These DEGs were mainly enriched in innate and adaptive immune responses, and inflammation, such as imune response-regulating cell surface receptor signalling pathway, tumour necrosis factor superfamily cytokine production (Figure S1A). Functional analysis of DEGs by direction showed that up-regulated DEGs were also enriched in biological processes associated with innate immune responses and inflammation, such as myeloid leukocyte activation and acute inflammatory response (Figure 3B). Notably, down-regulated genes were mainly enriched in immunological processes, especially T-cell immunity, such as T-cell differentiation and lymphocyte development (Figure 3C), suggesting complex immunomodulatory effects of GX19 infection.

Cardiac transcriptomic analysis identified 99 DEGs following GX19-6 infection (83 up-regulated and 16 down-regulated; Figure 3A). These DEGs were enriched in diverse functions, including immune response, steroid hormone secretion, lymphocyte proliferation, apoptotic process, hyperoxia, cell cycle, ATP metabolic process, and so on (Figure S1B). Functional analysis of DEGs by direction showed that up-regulated genes demonstrated significant enrichment in immune-related pathways, including humoral immune response and response to tumour necrosis factor, as well as apoptosis (such as hepatocyte apoptotic process), ATP metabolism (such as negative regulation of ATP metabolic process and mitochondrion organization), and oxygen-related processes (such as cellular response to oxygen levels) (Figure 3D). Intriguingly, down-regulated genes were predominantly associated with corticosteroid hormone secretion, regulation of endocrine processes, and modulation of apoptotic signalling pathways (Figure 3E), suggesting potential cardiac-endocrine crosstalk during viral infection.

Transcriptional profiling of brain tissue showed minimal gene expression alterations, with only 12 DEGs detected (11 up-regulated and 1 down-regulated; Figure 3A). These DEGs were enriched in only a few biological processes (Figure S1C). More sensitive Gene Set Enrichment Analysis (GSEA) uncovered subtle but coordinated changes in ribosomal biogenesis and protein translation pathways (Figure 3F), suggesting potential modulation of neuronal protein synthesis machinery following viral infection.

### Specific transcriptome changes of GX19-6

**Figure 4.**
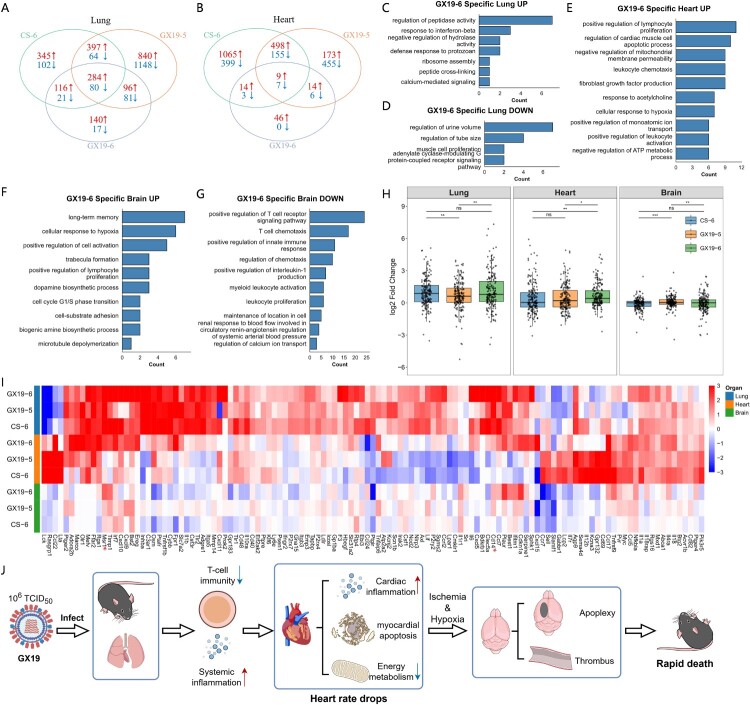
GX19-6 specific genes and functions relative to GX19-5 and CS-6. (A) and (B) refer to the Venn diagram of DEGs identified in the lung and heart, respectively, that were infected by GX19-6, GX19-5, and CS-6. (C)∼(E) refer to the enriched biological processes of GX19-6-specific DEGs in the mice's lung and heart compared to GX19-5 and CS-6. The GO terms were clustered, and each subpanel showed the number of GO terms in each cluster. (F) and (G) refer to the enriched biological processes of the top 100 GX19-6-exclusive up-- and down-regulated genes, respectively. (H) Comparison of fold changes of inflammation response genes in the mice's lung, heart, and brain between different viruses using the paired Wilcoxon rank-sum test. ns, no significant; *, *p*-value <0.05; **, *p*-value <0.01; ***, *p*-value <0.001. (I) Fold change of inflammation response genes in the mice's lung, heart, and brain after virus infections. (J) Diagram of a hypothesis about the rapid death of mice caused by the GX19 virus.

Next, we identified GX19-6-specific transcriptome changes by comparing DEGs identified in GX19-6 and those in GX19-5 and CS-6. In the lung, 157 GX19-6-specific DEGs were identified (140 up-regulated and 17 down-regulated) (Figure 4A). These DEGs were enriched in diverse functions, including proteolysis, peptidase activity, interferon response, and muscle contraction (Figure S2). Functional analysis of DEGs by direction showed that up-regulated DEGs were also enriched in diverse biological processes, including peptidase activity, interferon response, and negative regulation of hydrolase activity (Figure 4C), while the down-regulated DEGs were primarily associated with several biological processes that seem to be irrelevant to viral infections, such as tube size maintenance and muscle cell proliferation (Figure 4D). In the heart, 46 GX19-6-specific DEGs were identified, and all were up-regulated (Figure 4B). These DEGs were enriched in the immune system (such as lymphocyte proliferation), apoptosis (such as cardiac muscle cell apoptotic process), and energy metabolism regulation (such as negative regulation of mitochondrial membrane permeability, negative regulation of ATP metabolic process, and response to hypoxia) (Figure 4E). These transcriptional changes aligned with the more severe pathological manifestations observed in the heart of GX19-6-infected mice.

In the brain, we identified only two GX19-6-specific up-regulated DEGs (Figure S3), which were primarily associated with circadian rhythm regulation and cellular differentiation processes. To elucidate potential mechanisms underlying GX19-6-induced neuropathology, we performed focussed analysis of the top 100 GX19-6 exclusive up- and down-regulated genes. The up-regulated genes were associated with diverse functions such as long-term memory, cellular response to hypoxia, positive regulation of cell activation, and so on (Figure 4F), while the down-regulated genes were predominantly related to immune functions, particularly T-cell immunity (Figure 4G). This suggests that GX19-6 infection may suppress immune responses in the mouse brain.

Immune cells in tissues may have a large influence on the DEG analysis. Thus, we computationally inferred the immune cell compositions in mice's lungs, hearts, and brains using the bulk RNA-Seq data. As shown in Figure S4, in the heart and brain, only minor differences were observed between virus infection groups and PBS group; In the lung, the virus infection groups had higher ratios of monocytes and M1 macrophage, and a lower ratio of CD4 naive T cells compared to the PBS group, suggesting that the observed DEGs in the lung may be influenced by the immune cell composition. However, when targeting GX19-6, we observed overall similar immune cell compositions across all three tissues compared to both CS-6 and GX19-5. This similarity suggests that the immune cell composition may have a limited impact on the GX19-6-specific DEG analysis.

### Compensatory innate immune activation by GX19-6

Given the observed T-cell immunity suppression (Figure 3C & Figure 4G), we hypothesized compensatory innate immune activation. Accordingly, we analysed the fold changes of expression of inflammation-related genes in the lungs, heart, and brain after viral infections. As shown in Figure 4H&I, most inflammatory factors in GX19-6 virus infections were up-regulated in the lung and heart. The fold changes of inflammatory factors in GX19-6 infections were higher than those in GX19-5 and CS-6 in the heart and were comparable to those in CS-6 in the lung and brain. Some factors, such as Cd14 and Msr1 (marked by stars), showed significantly larger fold changes in GX19-6 infections than those in GX19-5 and CS-6 infections in all three organs. These results suggest systemic innate immune hyperactivation as a potential compensatory mechanism for T-cell suppression.

Then, we analysed the mechanisms underlying the evaluated expressions of inflammation-related genes. We firstly analysed the expression changes of three kinds of pattern recognition receptors (PRRs) after viral infections, including TLR (Toll-like receptor), RLR (RIG-I-like receptor), and NLR (NOD-like receptor) (Figure S5). In the lung, both GX19-6 and CS-6 showed significant up-regulations in the TLR and NOD-associated pathways compared to the PBS group. For the RLR, only GX19-6 showed some extent of up-regulation. In both heart and brain, three PRR-associated pathways did not show up-regulation in viral infections. In most cases, GX19-6 showed similar or lower fold changes compared to the other two viruses.

The high viral inoculum in virus infections of mice may lead to cell death, which can result in inflammation independent of PRRs. Thus, we compared the gene expression levels of four cell-death-related pathways, including apoptosis, ferroptosis, necroptosis, and pyroptosis, between three viruses in three tissues. As shown in Figure S6, for the apoptosis-associated pathway, all three viruses had no obvious up-regulations in the three tissues. For the ferroptosis and pyroptosis-associated pathways, GX19-6 showed the highest fold changes among the three viruses in all tissues, especially in the lung and heart. For the necroptosis-associated pathway, GX19-6 showed comparable (lung) or lower (heart and brain) fold changes than the other two viruses.

### Specific transcriptome changes of CS-6

We also identified CS-6-specific transcriptome changes by comparing DEGs identified in CS-6 and those in GX19-5 and GX19-6 (Figure 4A&B, Figure S3). In the lung, 447 CS-6 specific DEGs were identified (345 up-regulated and 102 down-regulated) (Figure 4A). These DEGs were mainly enriched in the immune system and response to viruses (Figure S7A). The up-regulated DEGs also showed significant enrichment in immune processes and response to viruses, such as regulation of immune effector process, regulation of interleukin-6 production, and response to viruses (Figure S7B), while no biological processes were enriched in the down-regulated DEGs. In the heart, 1464 CS-6-specific DEGs were identified (1065 up-regulated and 399 down-regulated) (Figure 4B). These DEGs were mainly enriched in adaptive immunity, cell cycle, inflammation, and response to viruses (Figure S7C). The up-regulated DEGs were enriched in the immune system, such as regulation of immune effector processes and regulation of T-cell activation (Figure S7D), while the down-regulated DEGs were enriched in the muscle system and development (Figure S7E). In the brain, 16 CS-6-specific DEGs were identified (6 up-regulated and 10 down-regulated) (Figure S3). They were enriched in only one biological process (defence response to viruses) (Figure S7F). The up-regulated DEGs were also mainly enriched in innate immunity and response to viruses, such as response to type 1 interferon, interferon-alpha production, and defence response to viruses (Figure S7G), while no enriched functions were observed for the down-regulated DEGs.

## Discussions

The severity of influenza virus infection is generally associated with viral load in the host. Here, we observed that the GX19 virus caused significantly greater damage in mice at high doses compared to low doses. Although GX19 exhibited much weaker replication in the lungs than the CS virus, with substantially lower viral titres, it led to faster mortality and more severe pathology in multiple organs. This suggests that GX19 may employ a novel mechanism to induce fatal outcomes in mice. Pathological analysis revealed that GX19-6 infection caused severe damage to the lungs, heart, and brain, which was more pronounced than that induced by GX19-5 or CS-6. Notably, brain damage in GX19-6-infected mice was particularly severe, with significant haemorrhage and oedema, indicating that rapid death may result from acute brain injury. Transcriptomic data showed that GX19-6 suppressed T-cell immunity in the lungs and brain while elevating systemic inflammation, possibly due to cell death (ferroptosis or pyroptosis). In the heart, increased inflammation and apoptosis were accompanied by reduced energy metabolism and weakened cardiac function. Hypoxic responses were observed in the heart, lungs, and brain (Figure 4E&F and Figure S6). Based on these findings, we propose the following hypothesis (Figure 4J): GX19 infections in the mice lung trigger excessive systemic inflammation, leading to cardiac inflammation, apoptosis, and metabolic dysfunction. This impairs heart function, causing cerebral hypoxia, thrombosis, and stroke, ultimately resulting in the rapid death of mice.

Previous studies have reported that influenza virus infection in the lungs can lead to damage in other organs such as the heart, kidneys, and brain [23-25]. However, these studies typically involved high-dose viral infections where the virus directly entered the bloodstream to affect multiple organs [26]. For example, research by Zheng et al. demonstrated that severe influenza infection could lead to viremia, allowing the virus to directly infect various organs and cause sepsis [27]. Alternatively, some neurotropic strains, such as A/Vietnam/1203/04 (H5N1), can invade the central nervous system through neural pathways [28]. In contrast, our study found that GX19 exhibited poor replication in the lungs, and no virus was detected in the heart and brain by TCID_50_ analysis (data not shown). This suggests that GX19 induces rapid mortality through a mechanism distinct from previously reported pathways. As outlined earlier, we propose that GX19 triggers inflammation-mediated lung-heart–brain axis dysfunction, ultimately leading to fatal outcomes without inducing a classic cytokine storm. However, the precise mechanisms by which pulmonary inflammation causes cardiac injury and subsequent cerebral haemorrhage remain unclear and warrant further investigation.

The dissociation between viral replication and pathogenicity observed with GX19 challenges conventional paradigms of influenza virulence and highlights the need to explore alternative pathogenic mechanisms beyond viral load-dependent models. Our findings suggest that certain influenza strains may cause severe disease through indirect, inflammation-driven multi-organ failure rather than direct viral cytopathic effects or classical cytokine storms. This novel pathogenic mechanism warrants further investigation into the specific viral factors responsible for triggering such disproportionate inflammatory responses despite limited replication, as well as the molecular pathways connecting pulmonary inflammation to remote organ damage. Understanding these alternative virulence mechanisms could lead to new therapeutic strategies targeting inflammation-mediated organ dysfunction in severe influenza cases.

## Conclusion

This study represents the first identification of a unique H3N2 influenza strain capable of inducing rapid mortality in mice despite demonstrating attenuated pulmonary replication. Through systematic pathological evaluation coupled with multi-organ transcriptomic profiling, we have elucidated a previously unrecognized pathogenic mechanism wherein the virus triggers fatal outcomes via a cascade of inflammation-mediated events along the lung-heart–brain axis, culminating in catastrophic cerebral injury. These findings not only expand our understanding of influenza virus pathogenesis beyond conventional replication-dependent models but also provide critical insights for developing targeted therapeutic interventions against severe influenza cases.

## Materials and methods

### Cell culture, viral propagation, and growth kinetics

Madin-Darby canine kidney (MDCK) cells were maintained in Dulbecco’s Modified Eagle’s Medium (DMEM; Invitrogen, Carlsbad, CA, USA) supplemented with 10% foetal bovine serum (FBS; Invitrogen), penicillin (100 U/mL), and streptomycin (100 μg/mL; Invitrogen). Two H3 subtype influenza virus strains – A/Environment/Guangxi/44461/2019 (GX19, H3N2) and A/Changsha/1000/2022 (CS, H3N8) [29] – were propagated in 9- to 11-day-old embryonated chicken eggs. Viral titres were quantified by determining the 50% tissue culture infectious dose (TCID_50_) in MDCK cells. For viral growth kinetics analysis, MDCK cells were infected with either GX19 or CS virus at a multiplicity of infection (MOI) of 0.001. Following infection, cells were incubated at 37°C in infection medium containing 2 mg/mL N-p-tosyl-L-phenylalanine chloromethyl ketone (TPCK)-treated trypsin (Sigma, St. Louis, MO, USA). Culture supernatants were harvested at 24, 48, 72, and 96 h post-infection (hpi), and viral titres were determined via TCID₅₀ assay on MDCK cells as previously described [30].

### Pathogenicity and replication kinetics of GX19 in mice

Specific-pathogen-free (SPF) female C57BL/6 mice (8–10 weeks old) were obtained from Vital River Laboratories (Beijing, China). Mice (n = 5 per group) were anesthetized with isoflurane and inoculated intranasally with 50 μL of either 10^5^ TCID_50_ of GX19 (H3N2), 10^6^TCID_50_ of GX19, or 10^6^ TCID_50_ of CS (H3N8). A control group (n = 5) received 50 μL of phosphate-buffered saline (PBS). Body weight and clinical signs were monitored daily for 14 days post-infection (dpi). Mice exhibiting >25% weight loss were humanely euthanized and recorded as fatalities. For tissue tropism analysis and viral replication analysis, groups of mice were intranasally challenged with 10^6^TCID_50_ of the respective viruses. At 1 and 4 dpi, three mice per group were euthanized, and tissues, including the nasal turbinate, trachea, lung, brain , heart, spleen, liver, eyes, kidney, and intestinal tissues, were collected. Viral titers in homogenized tissues were quantified by TCID₅₀ assay [30], with all lung tissues weighed prior to homogenization and the resulting TCID₅₀ values expressed as per gram of tissue. Virus detections were verified additionally by RT-qPCR assay. Briefly, RNA standards (10¹ – 10⁷ copies/μL) were prepared from M gene recombinant plasmids to generate a standard curve (R²≥0.99). Viral RNA was extracted using the RNeasy Mini Kit (Qiagen). TaqMan-based RT-qPCR targeting the influenza M gene was performed on a real-time PCR system: 42°C for 30 min, 95°C for 5 min, followed by 40 cycles of 95°C for 15s and 60°C for 30s. Viral copy numbers were calculated via the standard curve equation and adjusted for elution volume and dilution factor to obtain the original sample's viral load.

### Hematoxylin and eosin (H&E) staining

At 4 dpi, lung, heart, and brain tissues were collected from C57BL/6 mice and immediately fixed in 4% paraformaldehyde at 4℃ for 24 h. The fixed tissues were dehydrated in a graded ethanol series (70%, 80%, 90%, 95%, and 100%, 1 h each), cleared in xylene (twice, 15 min each), and embedded in paraffin. Paraffin blocks were sectioned at a thickness of 3∼4 μm using a rotary microtome (Leica RM2235, Leica Biosystems, Germany). One tissue section was randomly obtained for each mouse's lung, heart, and brain. Tissue sections were deparaffinized in xylene (twice, 10 min each), rehydrated through a descending ethanol series (100%, 95%, 90%, 80%, and 70%, 5 min each), and rinsed in distilled water. They were immersed in haematoxylin for 5 min, differentiated in 1% acid ethanol for 30 s, rinsed in running tap water for 5 min, and then counterstained with eosin for 2 min.

### Lung injury assessment

Microscopic images were captured using an Olympus BX53 microscope equipped with a DP74 digital camera at 20× magnification. Uniform settings for brightness, contrast, and thresholding were applied during image acquisition and analysis to ensure consistency across samples. Lung injury was semi-quantitatively assessed on H&E-stained lung tissue sections by two blinded pathologists based on three histological features: alveolar oedema, structural damage, and inflammatory cell infiltration. Each parameter was scored on a scale from 0 to 4, where 0 indicated no abnormality, 1 indicated minimal changes, 2 indicated mild changes, 3 indicated moderate changes, and 4 indicated severe or extensive damage. This results in a total injury score ranging from 0 to 12. For each H&E-stained section, at least five non-overlapping high-power fields (40×magnification) (ROI) were selected from representative regions, including the alveolar area, perivascular space, and bronchiolar surroundings, to ensure a comprehensive assessment of lung pathology [31].

### Cardiac injury assessment

Cardiac injury was assessed semi-quantitatively on H&E-stained heart tissue sections based on two key histopathological parameters: inflammatory cell infiltration and structural damage. For each parameter, severity was scored on a scale from 0 to 4. The total cardiac injury score ranged from 0 to 8. Histological evaluation was performed independently by two blinded pathologists to ensure objectivity and consistency. Sections were examined in at least five non-overlapping high-power fields (HPFs, 40× magnification) selected from representative regions of the left ventricular wall, interventricular septum, and subendocardial area to ensure comprehensive assessment of myocardial injury.

### Brain injury assessment

Brain tissue sections (4 μm thick) were stained with haematoxylin and eosin (H&E) according to standard protocols. Histopathological evaluation was independently performed by two blinded investigators under 40× objective magnification. Three key parameters were semi-quantitatively assessed within predefined regions of interest (ROIs): (1) Perivascular inflammatory cell infiltration, scored on a scale of 0–3 (0 = none; 1 = mild, scattered cells; 2 = moderate, partial or complete perivascular cuffing; 3 = severe, multilayered cuffing or diffuse infiltration), with the final score calculated as the mean from two representative ROIs; (2) Cerebral oedema, evaluated by interstitial sparsity and scored from 0 to 3 (0 = compact tissue; 1 = mild focal spacing; 2 = moderate diffuse spacing; 3 = severe vacuolization or rarefaction), excluding regions adjacent to large vessels or haemorrhage; and (3) Haemorrhage and vascular abnormalities, assessed by counting the number of haemorrhagic foci per section and determining the proportion (%) of vessels exhibiting wall thickening or red blood cell (RBC) extravasation, with at least 10 vessels evaluated per ROI.

### RNA extraction, library preparation, and RNA-Seq

Total RNA was extracted from homogenized lung, brain, and heart tissues, which were collected at 4 dpi, using the TRIzol reagent (Qiagen), and were purified using a RNeasy Plus Universal Mini kit (Qiagen) with DNase I digestion, according to the manufacturer’s instructions. The RNA integrity was verified using an Agilent 2100 Bioanalyzer (RNA Integrity Number, RIN≥7.0) (Table S4). The RNA quality was assessed using a NanoDrop spectrophotometer (A260/A280 ≥ 1.8. A260/A280 ≥ 2.0). The cDNA library was prepared using NEBNext® Ultra™ II RNA Library Prep Kit for Illumina (NEB) according to the manufacturer’s protocol. Briefly, mRNA was purified from 2 ug of total RNA using oligo (dT) magnetic beads. Divalent cations were used to fragment the purified mRNA into small pieces at 94℃ for 5 min; thereby priming bias was avoided when synthesizing the cDNA. The cleaved RNA fragments were used for double-stranded cDNA synthesis with random hexamer (N6) primers. The synthesized cDNA was subjected to end repair and a-Tailing processes before ligation of the adaptors. The end products were enriched by PCR to create the final cDNA library with sequences of approximately 300 bp. The libraries were analysed for size using Agilent 5200 (Agilent Technologies, Santa Clara, CA), and the concentration was determined using a Qubit™ 3 Fluorometer with the Qubit™ 1X dsDNA HS Assay Kit and Qpcr. The final DNA libraries were sequenced on the Illumina Novaseq 6000 platform (Illumina, San Diego, CA, USA) using V4 sequencing chemistry to generate approximately 30 million reads per sample.

### RNA-Seq data processing and differential gene expression analysis

Raw sequencing reads were trimmed for adapters and low-quality bases using fastp (v0.23.2) [32]. Clean reads were aligned to the Mus musculus reference genome (GRCm39) from NCBI using HISAT2 (v2.2.1) with default parameters [33]. Gene-level read counts were obtained using FeatureCounts (v2.0.1) [34]. To evaluate global transcriptomic variation across samples and to identify potential outliers or batch effects, principal component analysis (PCA) was performed using the prcomp function in R (v4.3.1). Raw count data were directly analysed using the DESeq2 package (v1.44.0) [35]. Differentially expressed genes (DEGs) were identified based on an adjusted *p*-value < 0.05 and an absolute log₂(fold change) ≥ 1. The DEGs of each virus in different tissues were obtained by comparing RNA-Seq data of virus infection samples with those of the PBS group.

### Gene function enrichment analysis

Gene Ontology (GO) enrichment analysis was conducted using the clusterProfiler package (v4.12.6) in R [36]. GO terms with *p*-value < 0.05 and q-value < 0.05 were considered significantly enriched. To further examine the functional relationships among enriched biological process (BP) terms, clustering was performed using rrvgo (v1.16.0), which groups GO terms based on their semantic similarity [37].

GSEA analysis of KEGG pathways was performed using the clusterProfiler (v4.12.6) R package.

Genes associated with several acute diseases in the lung, heart, and brain were curated from the DisGeNET database [38]. Using these disease gene sets, we performed Gene Set Variation Analysis (GSVA) with the R package GSVA (v1.52.3) [39] to transform the gene expression matrix into a disease signature enrichment score matrix. Subsequently, the differential activity of these disease signatures between experimental groups and PBS controls was assessed using linear models and empirical Bayes moderation implemented in the limma package (v3.64.3).

### Inflammation response genes and hypoxia response genes

Inflammation response genes were curated from the predefined HALLMARK_INFLAMMATORY_RESPONSE gene set in the Molecular Signatures Database (MSigDB). Hypoxia response genes were obtained from genes reported by *Ebersole et al., 2018*[40].

### Cell death-related genes and PRR-related genes

Genes associated with four distinct cell death pathways were derived from the MSigDB database, including apoptosis (HALLMARK_APOPTOSIS), pyroptosis (REACTOME_PYROPTOSIS), necroptosis (GOBP_NECROPTOTIC_SIGNALING_PATHWAY), and ferroptosis (GOBP_FERROPTOSIS). The genes related to pattern recognition receptors (PRRs) were sourced from the Gene Ontology (GO) database, specifically encompassing three key signalling pathways: the Toll-like receptor (TLR) signalling pathway (GO:0002224), the RIG-I signalling pathway (GO:0045089), and the NOD-like receptor (NLR) signalling pathway that merged genes from nucleotide-binding oligomerization domain containing 1 signalling pathway (GO:0070427) and nucleotide-binding oligomerization domain containing 2 signalling pathway (GO:0070431).

### Statistical analysis

All statistical analyses were conducted in R (version 4.3.1). Comparison of mice body weight loss or virus titres and comparison of injury scores between viruses were conducted with the Student's t-test using the *t.test()* function in R. Comparison of fold changes of inflammation response genes in mice lung, heart, and brain between different viruses was conducted with a paired Wilcoxon rank-sum test using the *pairwise.wilcox.test()* function in R. A *p*-value of less than 0.05 was considered statistically significant.

## Supplementary Material

SI.docx

## Data Availability

The raw sequence data reported in this paper have been deposited in the Genome Sequence Archive (Genomics, Proteomics & Bioinformatics 2021) in the National Genomics Data Center (Nucleic Acids Res 2022), China National Center for Bioinformation / Beijing Institute of Genomics, Chinese Academy of Sciences (GSA: CRA028348), which are publicly accessible at https://ngdc.cncb.ac.cn/gsa [41,42].
