# Supplementary material for: Low-replication influenza virus mediates high pathogenicity through an inflammation-driven lung–heart–brain axis in mice
Source: Emerg Microbes Infect. 2026 Jan 7;15(1):2608406. doi: 10.1080/22221751.2025.2608406 (PMC12781936; doi:10.1080/22221751.2025.2608406)
Supplement: SI.docx [file TEMI_A_2608406_SM4465.docx]

**Supplementary materials for**

**A low-replication influenza virus mediates high pathogenicity through an inflammation-driven lung-heart-brain axis in mice**

**Figure S1** The enriched biological processes of DEGs in lung, heart and brain after infection of GX19-6. The GO terms were clustered and each subpanel showed the number of GO terms in each cluster.


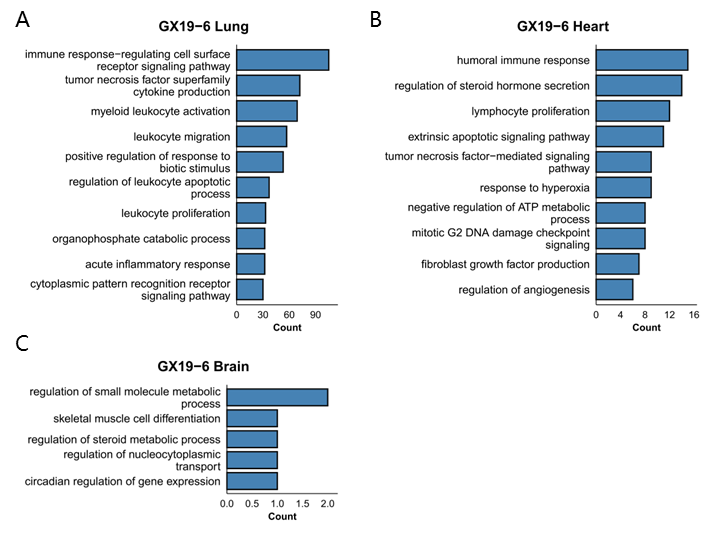


**Figure S2** The enriched biological processes of GX19-6 specific DEGs in the mice lung compared to GX19-5 and CS-6. The GO terms were clustered and each subpanel showed the number of GO terms in each cluster


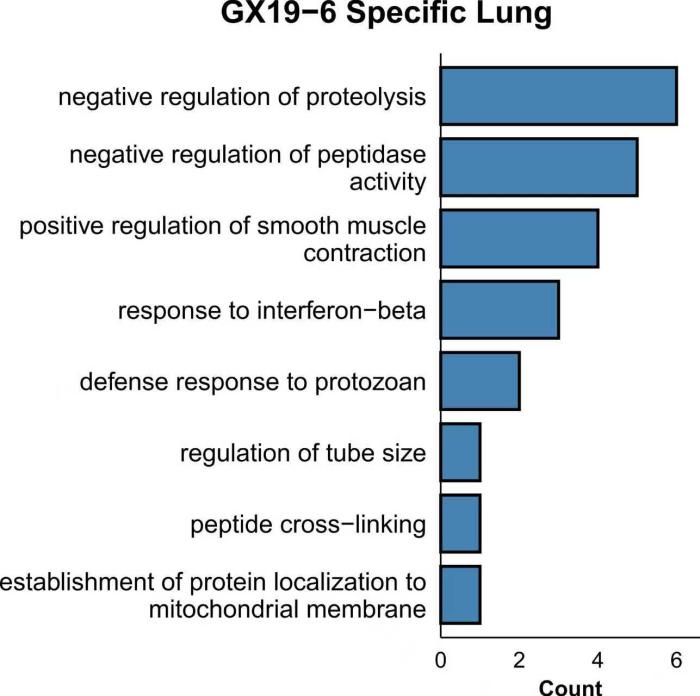


**Figure S3** Venn diagram of differentially expressed genes in the brain after infection with GX19-5, GX19-6 and CS-6.


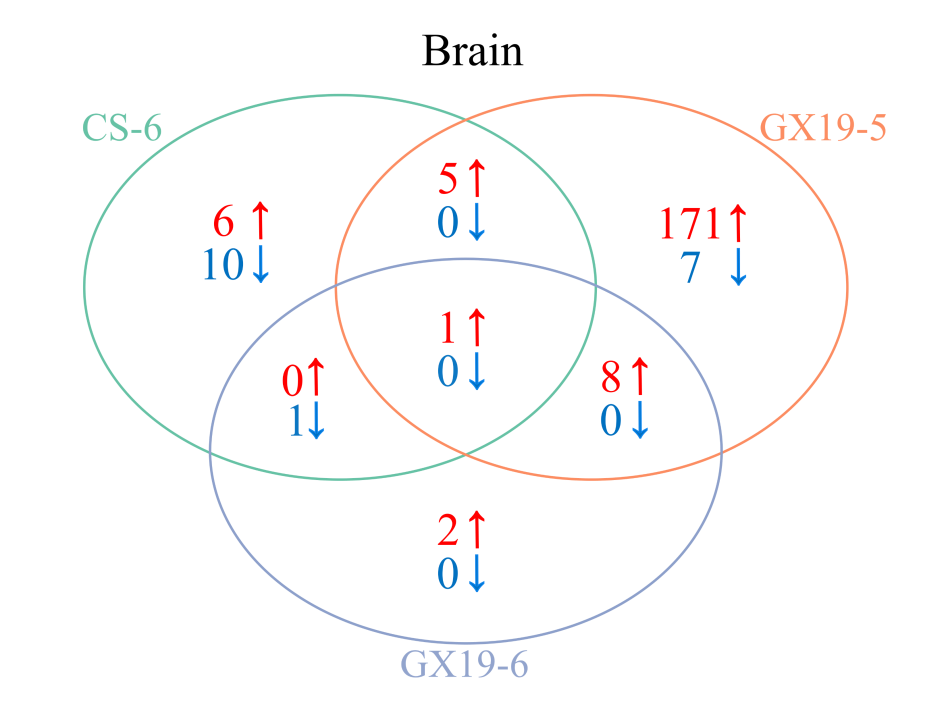


**Figure S4** The immune cell composition in lung, heart and brain after viral infections of CS-6, GX19-5 and GX19-6. They were inferred from the bulk RNA-Seq data using the CIBERSORTx method (https://cibersortx.stanford.edu).


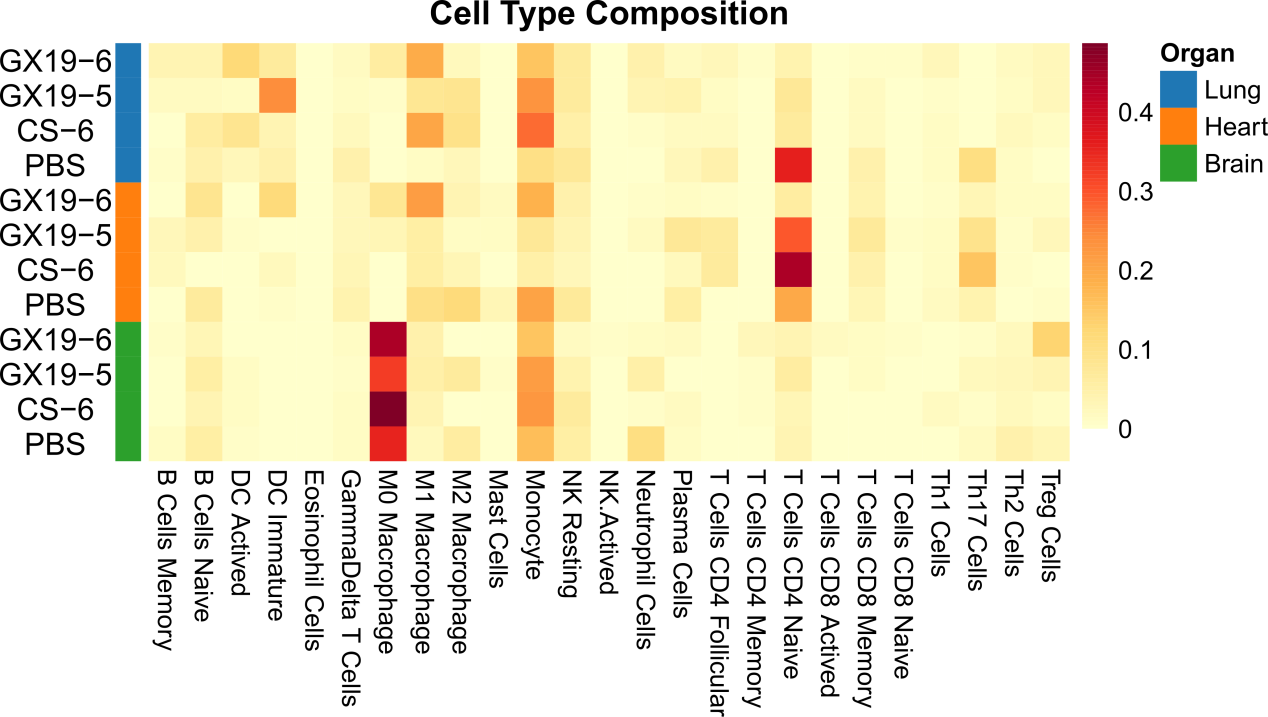


**Figure S5** The expression changes of three kinds of pattern recognition receptors (PRRs) including TLR (Toll-like receptor), RLR (RIG-I-like receptor) and NLR (NOD-like receptor) in lung, heart and brain after viral infections.


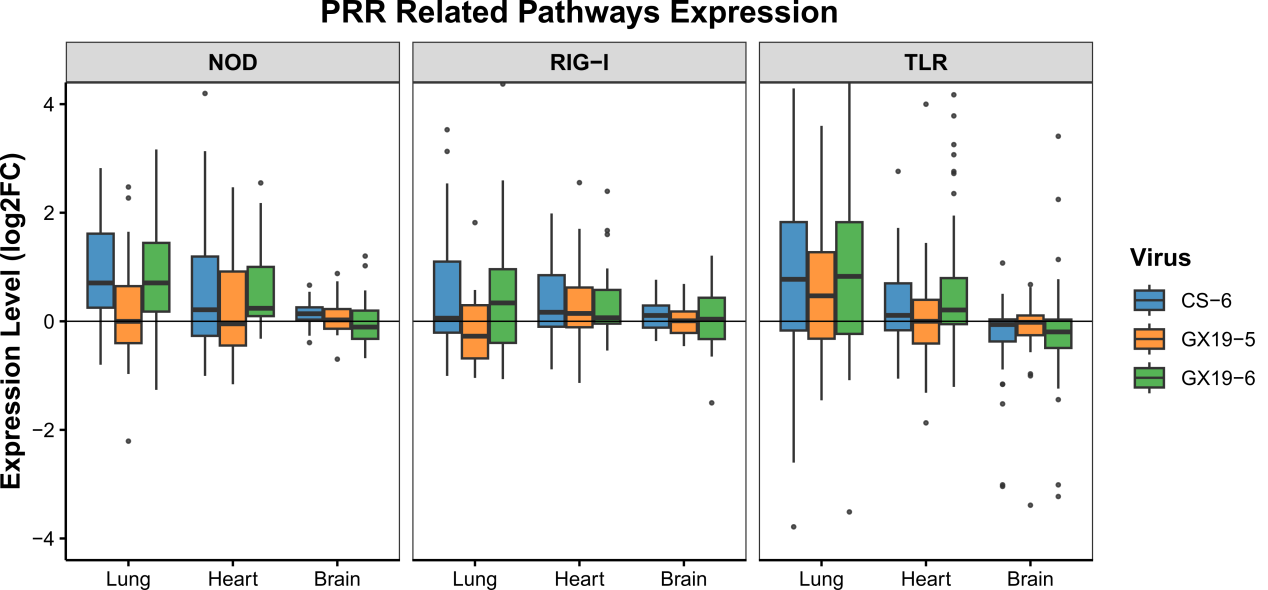


**Figure S6** The expression changes of four cell-death related pathways including apoptosis, ferroptosis, necroptosis and pyroptosis in lung, heart and brain after viral infections.


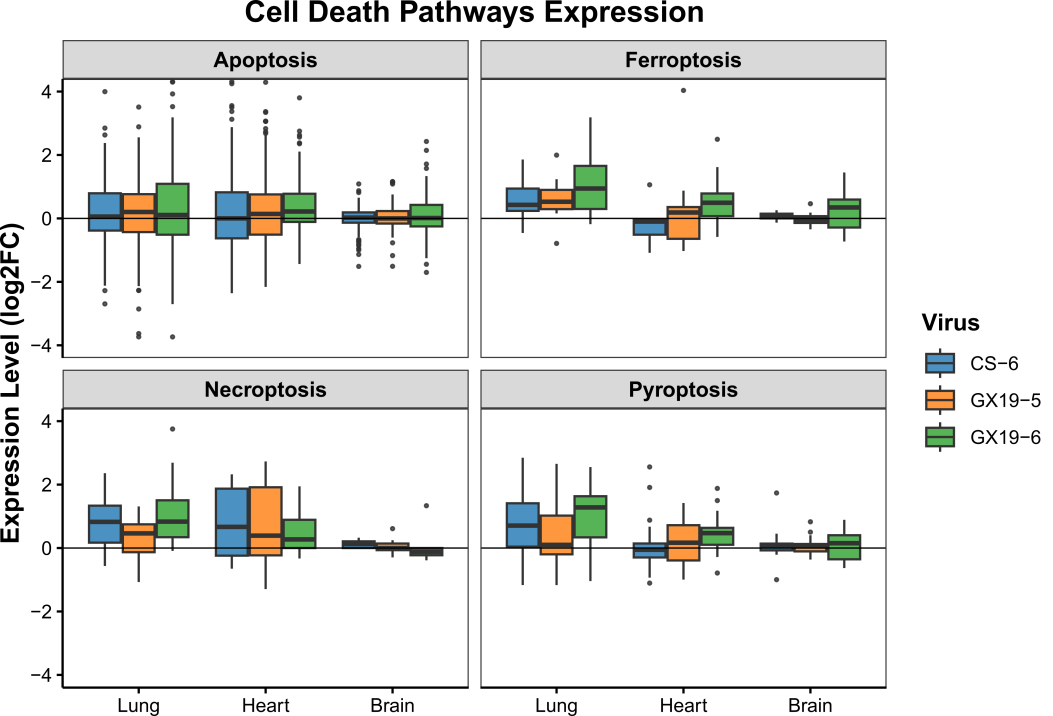


**Figure S6** Fold change in expression of hypoxia-related marker genes in the lungs, heart, and brain of mice infected with three influenza viruses (GX19-5, GX19-6 and CS-6) compared to the control group.


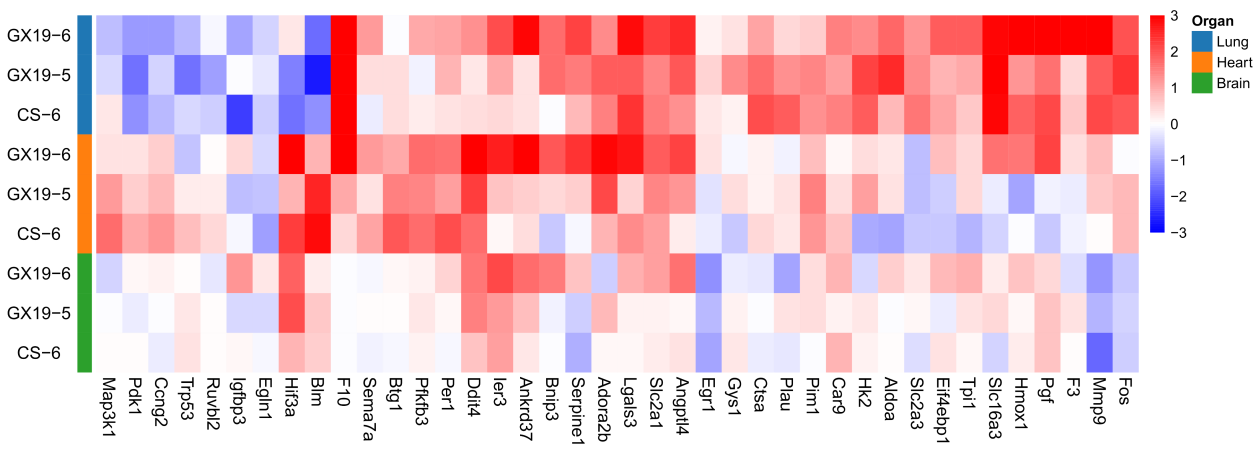


**Figure S7** CS-6 specific functions relative to GX19-5 and GX19-6. It shows the enriched biological processes of CS-6 specific DEGs in the mice lung (A), up-regulated DEGs in the mice lung (B), DEGs in the mice heart (C), up-regulated DEGs in the mice heart (D), down-regulated DEGs in the mice heart (E), DEGs in the brain (F) and up-regulated DEGs in the mice brain (G). The GO terms were clustered and each subpanel showed the number of GO terms in each cluster.


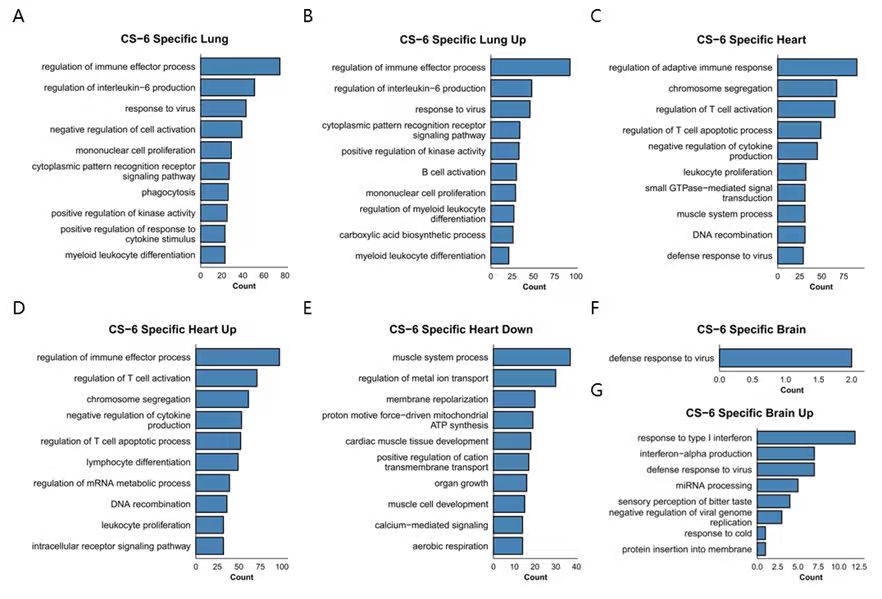


**Table S1.** Quantification of the extent of injury in mice lung from three aspects including alveolar edema, structural damage, inflammatory cell infiltration. Average and standard errors were provided.

| Group | PBS | CS-6 | GX19-5 | GX19-6 |
| --- | --- | --- | --- | --- |
| Alveolar edema | 0.83 ± 0.29 | 1.5 ± 0.50 | 2.17 ± 0.76 | 1.67 ± 0.29 |
| Structural damage | 0.17 ± 0.29 | 2.33 ± 0.29 | 0.83 ± 0.76 | 3.17 ± 0.76 |
| Inflammatory cell infiltration | 0.33 ± 0.58 | 1.67 ± 0.58 | 1.00 ± 0.87 | 2.00 ± 0.50 |
| Total | 1.33 ± 0.58 | 5.50 ± 0.50 | 4.00 ± 0.87 | 6.83 ± 0.76 |

**Table S2**. Quantification of the extent of injury in mice heart from two aspects: inflammatory cell infiltration, structural damage.

| Group | PBS | CS-6 | GX19-5 | GX19-6 |
| --- | --- | --- | --- | --- |
| Inflammatory cell infiltration | 0.50 ± 0.50 | 1.50 ± 0.00 | 1.00 ± 0.50 | 1.83 ± 0.58 |
| Structural damage | 0.33 ± 0.29 | 1.50 ± 0.50 | 0.67 ± 0.58 | 1.67 ± 0.29 |
| Total | 0.83 ± 0.58 | 3.00 ± 0.50 | 1.67 ± 0.29 | 3.50 ± 0.87 |

**Table S3**. Quantification of the extent of injury in mice brain from three aspects: perivascular inflammatory cell infiltration, cerebral edema, and cerebral hemorrhage

| Group | PBS | CS-6 | GX19-5 | GX19-6 |
| --- | --- | --- | --- | --- |
| Perivascular inflammatory cell infiltration | 0.00 ± 0.00 | 0.17 ± 0.29 | 1.00 ± 0.00 | 1.50 ± 0.00 |
| Cerebral edema | 0.33 ± 0.58 | 0.17 ± 0.29 | 1.00 ± 0.87 | 2.50 ± 0.71 |
| Cerebral hemorrhage | 0.00 ± 0.00 | 0.50 ± 0.50 | 0.67 ± 0.58 | 2.00 ± 0.71 |
| Total | 0.33 ± 0.58 | 0.83 ± 0.76 | 2.67 ± 0.29 | 6.00 ± 1.41 |

Note: GX19-6-Brain (n = 2), others (n = 3).

**Table S4**. The RIN scores for all samples used in the analysis.

| **Tissue** | **Virus** | **Sample ID** | **RIN score** |
| --- | --- | --- | --- |
| Lung | CS-6 | 976Lu | 9.4 |
|  |  | 979Lu | 9.2 |
|  |  | 997Lu | 9.6 |
|  | GX19-5 | 931Lu | 9.4 |
|  |  | 934Lu | 9.7 |
|  |  | 942Lu | 10 |
|  | GX19-6 | 933Lu | 10 |
|  |  | 935Lu | 9.5 |
|  |  | 941Lu | 10 |
|  | PBS | 947Lu | 9.6 |
|  |  | 949Lu | 9.5 |
|  |  | 950Lu | 10 |
| Heart | CS-6 | 976Hrt | 9.1 |
|  |  | 979Hrt | 8.6 |
|  |  | 997Hrt | 9.7 |
|  | GX19-5 | 931Hrt | 9 |
|  |  | 934Hrt | 9.6 |
|  |  | 942Hrt | 9.3 |
|  | GX19-6 | 933Hrt | 9.2 |
|  |  | 935Hrt | 9.6 |
|  |  | 941Hrt | 9.2 |
|  | PBS | 947Hrt | 9.1 |
|  |  | 949Hrt | 9.4 |
|  |  | 950Hrt | 9.2 |
| Brain | CS-6 | 976Br | 10 |
|  |  | 979Br | 9.4 |
|  |  | 997Br | 8.9 |
|  | GX19-5 | 931Br | 9.7 |
|  |  | 934Br | 9.7 |
|  |  | 942Br | 9.7 |
|  | GX19-6 | 933Br | 9.3 |
|  |  | 935Br | 9.1 |
|  |  | 941Br | 9.3 |
|  | PBS | 947Br | 9.4 |
|  |  | 949Br | 9.8 |
|  |  | 950Br | 9.9 |
